# What Instruments Shall I Carry?

**Published:** 1898-11

**Authors:** J. T. Codman

**Affiliations:** Boston, Mass.


### WHAT INSTRUMENTS SHALL I CARRY?

BY J. T. CODMAN, D.M.D., BOSTON, MASS.

A CALL came last night after midnight. A gentleman was suffering from toothache. He was a stranger at a hotel. Would I call on him and try to relieve him from pain? My first impulse was to say, No; if he wants my services let him come to me or wait until morning. But my second thoughts said, Wherefore am I a dentist, except it be to relieve pain? Is not that my highest professional duty? And I also remembered the injunction, Be careful in your entertainment of strangers, etc.

I dressed. It rained. Cars were not running. The messenger was gone; I could not question him. What was the trouble with the stranger? Had he an exposed pulp, simple periostitis, an abscess to be lanced, an old trouble from pent-up toxic matter, or new and suddenly violent inflammation of a pulp? And has the difficulty progressed so far that it is necessary to extract the tooth? I must be prepared for any emergency. If the gentleman were in my office, I could be sure of having at hand all appliances for any case. I judged it was probably pain from an exposed (diseased) pulp, and I gathered an assortment of instruments and appliances to take to his relief.

That was the only thing I could do. I could not take all in the office. I had no list made out. I must remember and recall all the things I have usually needed on similar calls. Why not have a list on hand? I will make one, and it will serve for future use. When I returned from my call I was more in favor of it, for I had left behind something it would have been best to have had by me.

This is the list I have made out, and as it may be useful to some professional brother (or sister) who has been awakened from a sound sleep some time, and required to put his ready wits to work searching among his multitudinous instruments for the right things to carry with him to the sufferer, I present it to the Journal.

EMERGENCY CALL LIST OF INSTRUMENTS AND APPLIANCES.

1 mouth-mirror (or two), to throw light.
1 explorer (or two), to search cavities.
1 large cutter (side)<br>1 large cutter (end) } to break edges and to open cavity.
1 (or two) long, smaller side cutter<br>1 (or two) long hoe excavator<br>1 short side cutter<br>1 short hoe excavator } called excavators, to clean and partly excavate cavity.
1 pair foil-tongs (always necessary).
1 water syringe (convenient).
1 knife file, or separating files (not always wanted).
1 half-round, medium-cut file, for shortening bite.
1 root instrument, for reaching into roots.

1 gum-lance. (Have a sharp knife in your pocket.)
6 small mouth-napkins, to dry with.
1 wad of cotton or absorbent fibre. (Do not forget it!)
1 spool floss silk or thread, to tie pledget in (sometimes).
1 pair scissors, to cut silk.
1 bottle sandarach varnish, to protect cotton filling.
1 bottle nerve-destroying paste, to kill pulps.
1 bottle 1, 2, 3 compound or cocaine, or any good obtundent.
1 small bottle toothache drops, to leave behind with patient.

On a chance of being obliged to extract an aching tooth, carry the following forceps:

1 pair upper wisdom.
1 pair under wisdom.
1 pair upper bicuspid.
1 pair under bicuspid.
1 pair upper central and cuspid.
1 pair under central and cuspid.
1 elevator. (Use your own kind.)

The wisdom forceps may, in an emergency, be used for all the molars; the bicuspid forceps for the canines. If not, use the central forceps for them and the remaining teeth.

Put all these implements into a small, neat hand-bag, with a clean towel, and, if room, add your thinnest office-coat, for comfort. Bring back all your instruments, etc., and your fee, if you can get it,—cash.

I have here made a list of about the smallest number of instruments, etc., that I would like to start out with at an unseasonable hour. In the daytime we may perhaps find what we have forgotten to take with us at a neighboring apothecary's store, or at some brother dentist's. If we are called to a private house and have left our cotton behind, we may find some there, or an old piece of cotton cloth, which may do, poorly, to form napkins of; but I modestly propose that this list of mine be copied and securely placed for reference where it can always be found. Each dentist may then subtract or add any other things to the list that he may prefer, or change some of them to those better suited to his fancy or individual taste.

